# The Strain on the Institutions

**Published:** 1915-11-20

**Authors:** 


### HOSPITAL EMPLOYEES AND ENLISTMENT.

### The Strain on the Institutions.

(From a Correspondent.)

No service, profession, or trade has sent to the war a larger proportion of its workers than the voluntary hospitals of Great Britain and Ireland; and for quality and usefulness of the units so sent the voluntary system may claim a high place. Our doctors and our nurses have occupied appropriate positions in the military system which it would have been difficult to fill from any other source. Our lay workers have freely offered themselves, chiefly as combatants, and if their services have not been used in the most economical way, they have played useful parts in the great business upon which the nation has embarked.

So freely has the voluntary system of hospitals offered its sons and daughters to the voluntary system of the Navy and Army that when Lord Derby's historical letter was issued it seemed hardly possible that the hospitals could denude the ranks of skilled workers still further; but, in company with all other branches of the community, greater and more important sacrifices are yet asked and expected.

### The Position at the London Hospitals.

At a recent meeting of hospital workers it was stated that in seventy hospitals in London (including the great hospitals) about 500 lay workers of military age were left. These included approximately 60 in the administrative department, 60 in the dispensaries, 65 in the works departments, 30 in the pathological departments and teaching sections; theatre and *x*-ray staffs accounted for 30; and the steward's department for 50. The rest were made up of engineers, stokers, porters, and sundry workers. Of these workers about 10 per cent. had already been rejected earlier or later in the war as unfit, and a few had been wounded and discharged. When the figures were compiled, a small number had enlisted and been returned for hospital duty until called up, but at the time of writing this number has probably been greatly added to.

The voluntary system is a consultative and specialised one. It is not every medical man who is suited to be a medical officer of a hospital, nor every dispenser who is an ideal hospital pharmacist. Secretaries have to be trained, however great their general abilities before they enter the service. A museum attendant, an *x*-ray assistant, or an out-patient porter is not made out of a non-skilled man in a few days or weeks. It is urged that substitutes must be found; but there are urgent reasons why the substitutes should be reasonably adequate, and it is certain that for some branches of our work substitutes cannot be found.

At the present time it is all the more important that skilled and knowledgeable people in the hospital world should occupy certain positions, for the financial aspect is such that it is vital that the right men should be at hand to find the money required for the work and, what is just as important, to spend it to the best advantage. Our income is exceedingly difficult to obtain, and of it can be said something that cannot be applied to the expenditure side of the account—that we know what is the minimum. The spending departments know neither the minimum nor the maximum sums required. Prices all have an upward tendency in some departments, such as drugs; no one can see where the rise is going to stop. In the ophthalmic hospitals the price of atropine is becoming prohibitive, and in the nerve hospitals bromides, the only specific for epileptic fits, has risen to such a figure that the position must sooner or later reflect upon a class of patients suffering from a complaint of a peculiarly distressing nature. These are but instances. We can eat margarine instead of butter, and do without bacon and new-laid eggs, but there are things which a hospital must have, whatever they may cost. Many officers are called upon to do more than routine duties; their experience and knowledge as buyers and distributors is of the greatest value.

### Round Pegs in Square Holes.

The Germans have taught us by their universal mobilisation a lesson which may not come altogether too late; they have recognised from the beginning the principle of the round peg in the round hole, and if their marvellous powers of organisation had in a moderate measure been possessed by our Government, there would be no question of able-bodied medical men being employed as administrators or registrars of medical hospitals, of ex-bank clerks and insurance clerks utilised as quartermasters, while voluntary hospital secretaries, pharmacists, and *x*-ray assistants are asked to lay aside their special talents and shoulder rifles.

The Government Departments are at cross-purposes in respect of hospitals. One Department begs them to receive more and more wounded and sick soldiers, while another is taking away the greater part of those who are competent properly to treat and look after the patients the hospitals are asked to provide for.

We understand that the asylums and mental hospitals have been asked to supply a list of indispensable workers of military age with a view to their exemption. In reference to voluntary hospitals Lord Derby has said that he cannot exempt skilled workers, and the best he can offer is that these shall be returned after enlistment and their cases shall receive consideration when the time comes for them to be called up; when, if they are still indispensable, they shall be relegated to a later group.

Their cases shall be considered. By whom? Presumably by the local tribunals that are to be set up. But a voluntary hospital belongs to too great a system to have its vital interests deliberated upon by a local tribunal, which probably will differ in opinions and procedure from the tribunal belonging to the next district. The question is not parochial; what is fair and expedient for one hospital should apply to all, and the matter is essentially one that must be dealt with by a Government Department.

We do not like the word "indispensable" in this connection. The authorities should ask each hospital to nominate the workers it is desirable to retain, and the outcoming nomination will not be merely in the interests of the hospital. The thing is much greater than this: the arrangement will be to the advantage of the whole civil population, of the sick and wounded soldiers in the care of the hospitals, and of the community at large. If the military authorities will take this step they may rely that the selection will be made in the fairest possible way, having regard not only to the interests we have mentioned but also to the needs of the military situation.

Except those who mourn their dead no one in this country truly knows what the words "suffering," "deprivation," and "discomfort" mean. A time may come when our sick poor and our wounded soldiers must put up with something short of the best that hospitals can give. But that day is not yet. At the present time there is no need to describe any hospital workers as indispensable, need only to remember that there is such a word as "economy," which means making the best use of available material.

God forbid that we shall see the day when the hospitals may be asked fairly to hand over the last few skilled workers who for economical reasons have been retained to a late hour. Then when every man must carry and use a weapon and all must go to man the wall the remaining hospital employees will cheerfuly lay down their task and play the new part. They are even ready now so to do if after full consideration of the position in all its aspects this duty is required of them.

